# Single neuron transient activity detection by means of tomography

**DOI:** 10.1186/1471-2202-12-S1-P297

**Published:** 2011-07-18

**Authors:** Carlos Aguirre, Pedro Pascual, Doris Campos, Eduardo Serrano

**Affiliations:** 1 GNB, Escuela Politécnica Superior, Universidad Autónoma de Madrid, 28049, Madrid, Spain

## 

Tomographic transforms [1] refers to a new kind of linear transforms that use a different approach than traditional transforms such as the Cohen’s Class or the Wigner distribution to obtain a representation of a signal in the time-frequency plane. The idea of tomography is to decompose the signal by using the eigenfunctions of linear combinations of operators, for example, time and frequency, time and resolution or time and conformal operator. Tomographic transforms has been used in the framework of quantum mechanics and for the analysis of reflectometry data [2]. Here we show that tomographic analysis can be also useful for the detection and characterization of transient components in neuronal signals.

We have applied the tomographic transform to both neuronal signals generated by a phenomenological model presented in [3] and to biological signals obtained from invertebrates. In Figure 1 A the output of a neuron with two different tonic spiking regimes produced by a different external current injection is depicted. In panel B, the values of the most significative coefficients of the Fourier transform are shown. In this case the Fourier transform detects the existence of different rhythms in the signal, but is not able to find the number of transient components. In panel C, the tomographic transform detects both the existence of different rhythms and the number of transient components of each rhythm in the neuron output signal.

## Conclusions

The tomographic transform shows itself as a robust method to disentangle components of similar frequency present at different time intervals and to detect different transitory rhythmic patterns present in a neuronal signal. This method could not only be useful to detect signal components localized in time, it can be also used as filtering tool by just preserving the most significant values of the tomographic transform, in a similar way as in the Fourier transform but with the advantage of preserving time information.

**Figure 1:**
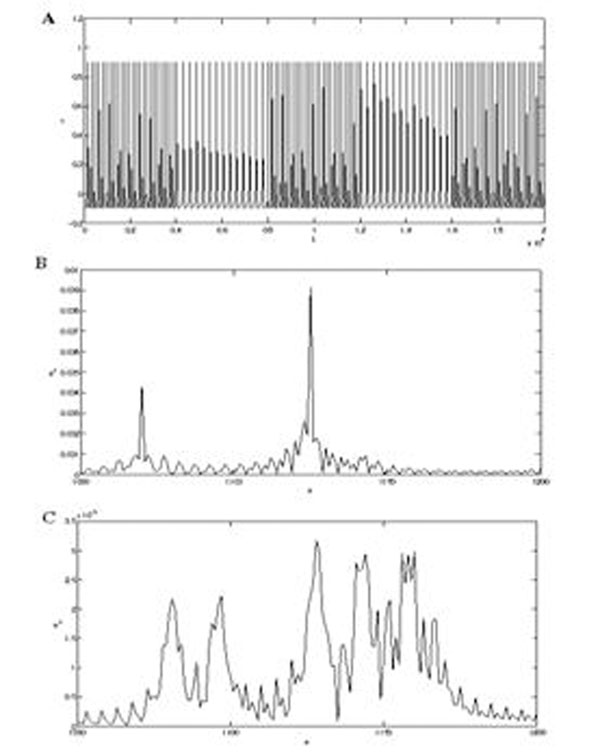
**A.** Neuronal Signal with transient behaviour. **B.** Fourier Transform. **C.** Tomographic Transform.
